# Plant Byproducts as Part of Edible Coatings: A Case Study with Parsley, Grape and Blueberry Pomace

**DOI:** 10.3390/polym13152578

**Published:** 2021-08-03

**Authors:** Alexandra Tauferova, Matej Pospiech, Zdenka Javurkova, Bohuslava Tremlova, Dani Dordevic, Simona Jancikova, Karolina Tesikova, Michal Zdarsky, Tomas Vitez, Monika Vitezova

**Affiliations:** 1 Department of Plant Origin Food Sciences, Faculty of Veterinary Hygiene and Ecology, University of Veterinary Sciences Brno, Palackeho tr. 1946/1, 612 42 Brno, Czech Republic; tauferovaa@vfu.cz (A.T.); pospiechm@vfu.cz (M.P.); javurkovaz@vfu.cz (Z.J.); dordevicd@vfu.cz (D.D.); jancikovas@vfu.cz (S.J.); tesikovak@vfu.cz (K.T.); zdarskym@vfu.cz (M.Z.); 2 Department of Experimental Biology, Faculty of Science, Masaryk University, Kamenice 5, 625 00 Brno, Czech Republic; tomas.vitez@mendelu.cz (T.V.); vitezova@sci.muni.cz (M.V.); 3 Department of Agricultural, Food and Environmental Engineering, Faculty of AgriSciences, Mendel University in Brno, Zemedelska 1, 613 00 Brno, Czech Republic

**Keywords:** biodegradability, sensory, scanning electron microscopy, CIE*Lab*, plant extracts

## Abstract

Studies dealing with the development of edible/biodegradable packaging have been gaining popularity since these commodities are marked as being ecofriendly, especially when byproducts are incorporated. Consequently, this study aimed at the development of chitosan-based coatings with plant byproducts. Their sensory properties, colour attributes, occurrence of cracks in microstructure and biodegradability were analysed. Coatings containing grape and blueberry pomace had statistically significantly (*p* < 0.05) higher levels of colour intensity. Coating samples were characterised by lower aroma intensity (3.46–4.77), relatively smooth surface (2.40–5.86), and low stickiness (2.11–3.14). In the overall hedonic evaluation, the samples containing parsley pomace in all concentrations and a sample containing 5% grape pomace achieved a statistically significantly (*p* < 0.05) better evaluation (5.76–5.93). The lowest values of the parameter ΔE2000 were recorded for the sample containing 5% parsley pomace (3.5); the highest was for the sample with 20% blueberry pomace (39.3). An analysis of the coating surface microstructure showed the presence of surface cracks at an 80 K magnification but the protective function of the edible coating was not disrupted by the added plant pomace. The produced samples can be considered to have a high biodegradability rate. The results of our experimentally produced coatings indicate their possible application on a commercial scale.

## 1. Introduction

Biodegradable edible coatings have been widely studied recently as they represent a sustainable and environmentally sound solution for primary packaging in the food industry. They can act as a barrier against microbial contamination, decrease the rate of oxidation, prevent or decrease the rate of moisture loss and other types of physical deterioration [1,2,3]. Barrier properties of edible coatings in the sense of physical coating integrity and cracks occurrence are often studied by means of scanning electron microscopy [4,5,6]. As they are in the closest contact with the food, their significant advantage is their ability to protect the food product in a non-toxic way. If antioxidant ingredients are included, they can further prolong the shelf life and enhance the nutritional value of a packaged food item by migrating such bioactive compounds from the coating [7,8]. Polyphenols are one of the main categories of commonly used bioactive agents [9,10,11]. Recent research studies showed new possibilities for how to isolate potent natural antioxidants or how to combine phenolic antioxidants with other functional ingredients to increase the efficiency of the manufacturing process in the food industry [12,13]. The use of bioactive compounds from various food industry byproducts is an effective strategy to decrease both the environmental impact and production costs [14,15].

Considering all these facts, an edible coating containing potent antioxidants derived from byproducts could be of great value for the food industry, as long as the particular coating is accepted by consumers with regard to its sensory properties. Therefore, it is essential to measure the acceptability of produced novel coatings for consumers. The attributes of the edible coating should not interfere with the sensory attributes of the food product [16]. 

Although there is still a gap between food packaging materials in the field of research and on the commercial scale, many biodegradable materials are being increasingly considered for food packaging production [17]. These include protein-, lipid- and polysaccharide-based coatings. To improve the natural properties of these polymers, additional functional ingredients or processing technologies, including nanotechnology among others, have been proposed [18]. In polysaccharide-based edible packaging materials, chitosan is one of numerous edible polymer options frequently used in the manufacturing of edible coatings [5,6,7,9,11]. Chitosan is the deacetylated form of chitin—the second most abundant polysaccharide in nature after cellulose [19]. The inclusion of chitosan has great potential as it shows high antimicrobial activity and compatibility with other biopolymers and active agents [20]. Table 1 shows a brief review of recently produced chitosan-based coatings, indicating the activities and the food matrix. 

Chitosan based edible/biodegradable films showed significant potential in the food industry, extending the shelf life of postharvest fruits, such as in the work of Jiang et al. [21], where chitosan coatings (both low molecular weight and high molecular weight) postponed the storage period of blueberry fruits. The loss of antioxidant capacity is the reason for the shorter storage period. Guava fruits immersed in chitosan coatings showed a reduced loss of antioxidant capacity during storage (20 days of low temperature storage) [22]. Chitosan coatings also showed their effectiveness at room temperature in concentrations of up to 3%. It was found that edible chitosan coatings prolonged the quality attributes of mango during storage at room temperature, affecting starch degradation rate and mitochondrial respiration [23]. There are also differences between coatings made from low, medium and high molecular weight chitosan. The study of Drevinskas et al. [24] showed that high molecular weight chitosan had a more highly positive influence on the shelf life of two kiwi cultivars (Sentiabrskaya and Anykšta cultivars) in comparison with VIR2 kiwi cultivar. Hard-green mangoes stored at room temperature and relative humidity of 60% to 70% during 2 weeks and immersed in the composite coating of chitosan (gallic acid:acetic acid) had lower decrease in acidity and vitamin C content. The using of composite coating resulted in better flesh firmness than in untreated hard-green mangoes [25]. Chitosan solution (1%) combined with spermidine (0.1 ppm) showed antibacterial properties against *Colletotrichum gloeosporioides* on mango fruits; the solution of chitosan and spermidine also improved the firmness and deterioration processes were delayed [26]. Fresh cut apples represent a very vulnerable commodity with a short shelf life. Chitosan with carboxy methylcellulose sodium were used as a coating for fresh cut apples, positively affecting the weight loss, firmness, and browning [27]. Nanocrystals of cellulose in combination with chitosan positively affected (delayed ripening) pear during storage at ambient temperature and cold storage [28].

**Table 1:** A brief review of chitosan-based coatings applied on fruits.

References	Chitosan	Food Matrix	Results
[21]	- Low molecular weight chitosan viscosity of 50 mPa·s and degree of deacetylation 91.0% - High molecular weight chitosan viscosity of 423 mPa·s and degree of deacetylation 95.6%)	Blueberry (*Vaccinium ashei* Reade)	Chitosan coating to blueberry positively effected the changes of weight loss, firmness, total phenolics and anthocyanins, same as storage time increased.
[22]	- Chitosan (low molecular weight, 75% deacetylated)	Guavas (cv *Allahabad safeda*)	Samples coated with chitosan and enriched with pomegranate peel extract were effective in maintaining the overall fruit quality.
[23]	- Chitosan (95–98% deacetylated)	Mango (*Mangifera indica* L.)	Chitosan delayed postharvest changes: climacteric peak, water loss and firmness.
[25]	- The composite of chitosan: gallic acid: acetic acid (2:1:1)	Hard-green ‘Hindi-Besennara’ mangoes	The decay and weight loss of fruits immersed in chitosan composite were slowed.
[26]	- 1% chitosan solution combined with 0.1 ppm spermidine	Mango (*Mangifera indica* L.)	Inoculated mango fruit coated with 1% chitosan and 0.1 ppm spermidine showed the lowest fungal decay.
[24]	- Low molecular weight chitosan - Medium molecular weight chitosan - High molecular weight chitosan	Kiwi fruit (*Actinidia kolomikta*)	High molecular weight chitosan had better positive effect on the shelf life of kiwi fruit cultivars.
[27]	- Chitosan (deacetylation degree ≥ 85%) with carboxy methylcellulose sodium (viscosity: 300–800 mPa.s)	Fresh cut apples	The coating positively affected weight loss, firmness, and anti-browning.
[28]	- Chitosan (97% degree of deacetylation) reinforced by cellulose nanocrystal	Pear fruit (*Pyrus communis* L.)	Chitosan coating reinforced by cellulose nanocrystal (5%) postponed chlorophyll degradation prevented internal browning and retained fruit firmness.

Blueberries have long been valued for their high levels of phenolic compounds and have the highest antioxidant capacity among traditional fruits and vegetables [29]. They contain high amounts of chlorogenic acid, which has been noted for its antioxidant properties and numerous flavonols, whose profiles differ between individual cultivars [30]. Grapes contain high amounts of phenols, flavonoids and anthocyanins with high antioxidant potential [31]. Anthocyanins are the characteristic pigments of grape berries and malvidin derivatives are the most abundant components, accounting for more than 68% of total anthocyanins in table grapes. Grape pomace is a high-quality biodegradable residue of the wine industry with a high potential to be used for further antioxidant extraction as substantial quantities of antioxidants remain in grape seeds, skins and stalks [32,33]. Parsley is a medicinal plant with various proven pharmacological properties including antioxidant, antibacterial and antifungal activities. Phenolic compounds, particularly flavonoids (such as apigenin-7-apiosylglucoside (apiin) and isorhamnetin-3-O-hexoside) are dominant compounds of parsley [34]. Other active compounds of parsley are essential oil components (especially myristicin and apiol), coumarins and furocoumarins [35].

In light of the above-mentioned information, the goal of this study was to develop bioactive edible coatings based on chitosan incorporated with three different concentrations of parsley, grape and blueberry pomace extracts, defined as a low-cost source of bioactive ingredients. Experimentally produced edible coatings were characterised by the following analyses: sensory analysis (quantitative descriptive analysis and hedonic analysis); colour parameters; scanning electron microscopy; and biological oxygen demand. Sensory and colour analyses allowed the evaluation of the real time consumers’ preferences and acceptance; scanning electron microscopy showed the microproperties of the experimentally produced packaging; biological oxygen demand (BOD) values showed the biodegradable potential of the packaging.

## 2. Materials and Methods

### 2.1. Materials

Plant raw materials used in this study to produce extracts for the purpose of preparing edible coatings were purchased at the local market in Brno, Czech Republic. These were specifically parsley (*Petroselinum*), grown in the Czech Republic; blueberries (*Vaccinium myrtillus* L.), grown in Spain; and seedless red grapes (*Crimson*), grown in Chile. Low molecular weight chitosan, as well as other chemicals used in the analyses, were purchased from Sigma-Aldrich (St. Louis, MO, USA).

### 2.2. Preparation of Extracts

Firstly, the above-mentioned plant raw materials were juiced and byproducts (parsley, blueberries and grapes pomaces) were obtained in the form of pulp and husk. These byproducts were used to prepare extracts. Subsequently, 10 g of the byproduct were weighed into a beaker and poured into 100 mL of hot distilled water (100 °C). After infusing for 10 min, the extract was filtered and used to produce edible coating.

### 2.3. Preparation of Edible Coatings

The edible coatings were prepared using the modified method according to Jancikova et al. [36]. Firstly, 1.5 g of low molecular weight chitosan was weighed into a 250 mL beaker and subsequently dissolved in 1% lactic acid (the amount of lactic acid solution was variable depending on the amount of the extract). For the preparation of the coating without extracts as a control, 135 mL of 1% lactic acid was used. The samples were then transferred to magnetic stirrers and stirred for 15 min (50 °C, 500 rpm). The plant extracts were then added at 3 different volume concentrations (5%, 10%, 20%) (the concentrations according to Jancikova et al. [3]) and the samples were stirred for an additional 5 min. Glycerol (0.75 mL) was added as a plasticizer. Afterwards, the coating solution was poured into 150 mm diameter Petri dishes and left to dry for 48 h. The composition of the edible coatings and related abbreviations are presented in Table 2.

### 2.4. Sensory Analysis of Edible Coatings

Sensory analyses were performed at the Department of Plant Origin Foodstuffs Hygiene and Technology of Faculty of Veterinary Hygiene and Ecology, University of Veterinary and Pharmaceutical Sciences Brno, Czech Republic. Both quantitative descriptive analysis and hedonic testing of prepared edible coatings were performed. The sensory analyses were performed in complete block design. Samples in the form of strips of edible packaging with approximate dimensions of 6 cm × 2 cm were presented in random order on clear plastic Petri dishes identified by 3-digit numerical codes in a monadic sequential presentation scheme. At the same time, an additional survey of the probability of the prepared edible coatings being purchased if used with certain groups of food products was carried out. 

#### 2.4.1. Quantitative Descriptive Analysis

A quantitative descriptive analysis of prepared edible coatings was performed by a trained panel consisting of academic staff of the Department of Plant Origin Food Sciences. The average age of the 12 panellists was 33.5 years. A panel discussion on the most cited descriptors of edible packaging [37,38,39,40,41] was performed in order to select those that best characterize the product and to eliminate those that were not perceived by most panellists. After the discussion and selection of the descriptors, a training session on the selected edible coating descriptors’ intensity scale preceded the quantitative descriptive analysis of the samples. Descriptors including colour intensity, surface character (smooth/rough), aroma intensity, and stickiness were evaluated. All attributes were quantified using a 9-point category ordinal scale with described extremes from 1 (no perception) to 9 (the highest intensity). The quantitative descriptive analysis was repeated three times.

#### 2.4.2. Hedonic Analysis

Moderately trained panellists (*n* = 55; average age 29.2 years) consisting of students and employees of the Faculty of Veterinary Hygiene and Ecology were recruited for the hedonic analysis. Pleasantness of appearance, aroma, texture and overall pleasantness were evaluated (1 × evaluation) using the 9-point category ordinal hedonic scale. (1 = dislike extremely, 5 = neither like nor dislike, 9 = like extremely). 

##### Purchase Probability Evaluation

The hedonic analysis also included a purchase probability evaluation for each group of food packed in the coatings produced for this study. A 5-point scale was used to evaluate the purchase probability: 1 = definitely would not eat the commodity in the analysed coating; 2 = probably would not eat the commodity in the coating; 3 = not sure whether to eat the commodity in the coating; 4 = probably would eat the commodity in the coating; 5 = certainly would eat the commodity in the analysed coating [37].

### 2.5. Measuring the Colour Parameters of the Edible Coating

The samples were placed on 150 mm diameter Petri dishes. Digital images of all experimental samples were acquired by a computer vision system. The images were taken under standard light conditions using 2 OSRAM DELUX L—1 × 18 W (OSRAM GmbH, Munich, Germany) lamps in a dark room. A Canon EOS 600D camera (Canon, Tokyo, Japan) mounted on a tripod (Fomei CS 920, Hradec Králové, Czech Republic) was used to take the images against a white background. The shooting was conducted in the Manual Mode, with an exposure time of 1/40, aperture F 5.6, image size L, sensitivity ISO 100 [42]. Ten images of each sample were obtained.

The images were processed by using the Nikon Imaging Software NIS-Elements BR 4.13.04 (Japan). The same region of interest (ROI) was selected for evaluating each image in the NIS-Elements. The colour characteristics—MeanRed, MeanGreen and MeanBlue—were measured, which were then converted to CIEL*a*b* space, where L* stands for lightness, a* indicates the position on red–green axis and b* on yellow–blue axis. ΔE as the colour difference between edible coatings with added extracts and control sample was calculated using CIE ΔE2000 equation [43,44].

### 2.6. SEM Evaluation of the Surface of Edible Coatings

The evaluation of the surface of the edible coating was performed after the gel formation on the conductive disc so as to minimise surface changes caused by bending or refraction during handling. For the index of refraction analysis, the edible coating was frozen with liquid nitrogen and mechanically broken. The fragments were stuck on carbon double-sided adhesive tape.

The samples were scanned with a MIRA3 TESCAN microscope (TESCAN ORSAY HOLDING, a.s., Brno, Czech Republic) at a voltage of 5.0 kV. Each sample was scanned three times. Images displaying a surface distortion were analysed using two micrographs obtained for each sample. This means that each sample was analysed at 6 randomly selected locations. The micrographs reported showed a magnification of 8 K, 80 K, and 800 K. A magnification of 25 K was used to view the images displaying the index of refraction of the coating.

### 2.7. Evaluation of the Edible Coating Biodegradability by Mixed Culture

Biological oxygen demand (BOD) values were determined by the aerobic system OxiTop^®^ (WTW, Weilheim, Germany) according to OECD guideline 301 F: Manometric Respirometry. A sample of edible coating in a mineral medium was inoculated (activated sludge) and incubated under aerobic conditions in the dark. The stock solutions for the mineral medium were prepared according to OECD guideline 301. Activated sewage sludge used in the experiments was taken from an activation tank at the wastewater treatment plant Modřice, 513,000 PE, Brno, Czech Republic. A measured volume of inoculated mineral medium (15 mL of sewage sludge and 28.5 mL of mineral medium), containing a known amount of the edible coatings (0.1 mg ± 0.0050 mg) as the nominal sole source of organic carbon, was stirred in a closed flask at a constant temperature 20 ± 1 °C. The tests were terminated when the biodegradation curve had reached a plateau for at least three determinations. The consumption of oxygen is determined by measuring the change in pressure in the flask. Evolved carbon dioxide is absorbed in sodium hydroxide. The amount of oxygen taken up by the microbial population during biodegradation of the test substance (corrected for uptake by blank inoculum, run in parallel) is expressed as a BOD in mg/L/g_dw_. The analysis was performed in triplicate.

### 2.8. Statistical Analysis

As the experimental design in the sensory analyses, a complete block design was used. Statistical analyses of sensory data were performed using the SensoMineR package for R software (The R Foundation for Statistical Computing, Vienna, Austria). Principal component analysis (PCA) was selected for the sensory data evaluation. The resulting colour parameters were statistically evaluated using the Unistat Tukey-HSD test procedure. Spearman’s correlation coefficient was used to express non-parametric correlations between sensory descriptors and colour parameters obtained by measuring instruments. A k-sample comparison of variances test (XLSTAT, Addinsoft, FR, version 2021) with a significance level α = 0.05 was used for the statistical processing of the total area and distance of cracks obtained by SEM. Outlying values were removed before the analysis by means of the Grubbs’ test of outliers (*p* < 0.05).

## 3. Results and Discussion

### 3.1. Results of the Sensory Analysis of Edible Coatings

The results of the sensory analysis are shown in two types of graphs: score plots for the mean points and variables factor maps. By score plots, difference vs. similarity between individual analysed samples is visualized. Variable factor maps visualize the relation between the principal components and the evaluated descriptors. 

**Figure 1:**
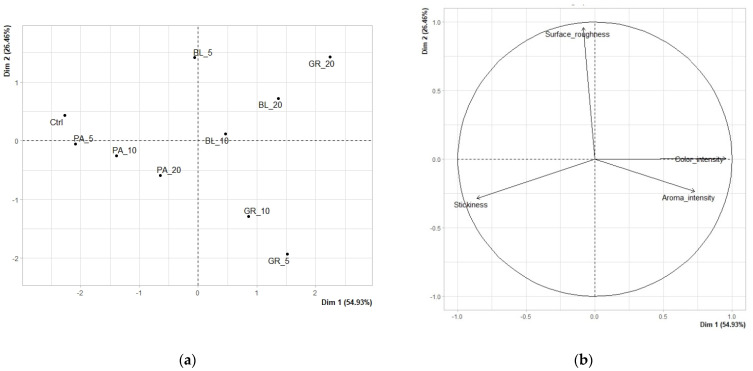
The results of PCA of quantitative descriptive analysis of edible coatings: (**a**) Score plot for the mean points (BL_5, edible coating with 5% of blueberry extract; BL_10, edible coating with 10% of blueberry extract; BL_20, edible coating with 20% of blueberry extract; GR_5, edible coating with 5% of red grape extract; GR_10, edible coating with 10% of red grape extract; GR_20, edible coating with 20% of red grape extract; PA_5, edible coating with 5% of parsley extract; PA_10, edible coating with 10% of parsley extract; PA_20, edible coating with 20% of parsley extract; Ctrl, control = edible coating without added extract). (**b**) Variables factor map.

**Figure 2:**
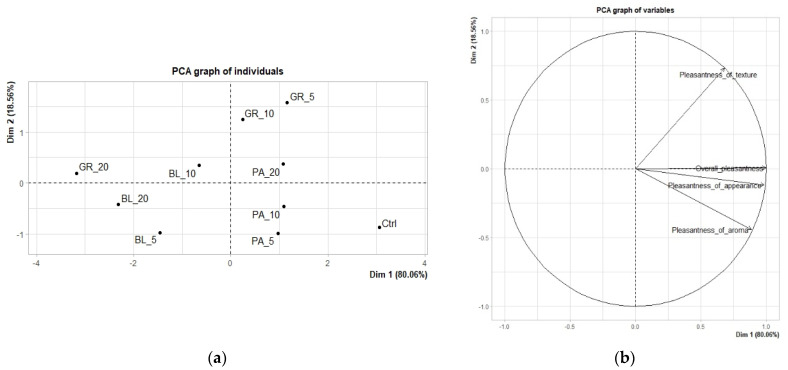
The results of PCA of hedonic analysis of edible coatings: (**a**) Score plot for the mean points (BL_5, edible coating with 5% of blueberry extract; BL_10, edible coating with 10% of blueberry extract; BL_20, edible coating with 20% of blueberry extract; GR_5, edible coating with 5% of red grape extract; GR_10, edible coating with 10% of red grape extract; GR_20, edible coating with 20% of red grape extract; PA_5, edible coating with 5% of parsley extract; PA_10, edible coating with 10% of parsley extract; PA_20, edible coating with 20% of parsley extract; Ctrl, control = edible coating without added extract). (**b**) Variables factor map.

The principal component analysis (PCA) emphasized the differences between the experimenally produced edible coatings since the distribution of samples in score plots (both quantitative descriptive and hedonic analysis) indicated dependence on extract concentrations and extract type (Figure 1 and Figure 2). These findings are important due to the fact that sensory properties, as the most important factor for the consumers’ acceptance, can be affected by the proper choice of plant extract.

#### 3.1.1. Quantitative Descriptive Analysis

Figure 1 shows the results of the quantitative descriptive analysis of the edible coatings. Both the location of each sample on the map of samples (Figure 1a) and the statistically significant (*p* < 0.05) differences between the edible coatings shown by Table 3 make it clear that the samples of edible coatings differed in many statistically significant aspects. The edible coatings containing grape and blueberry pomace in all concentrations (with the exception of the lowest 5% concentration of blueberry pomace) had statistically significantly (*p* < 0.05) higher levels of colour intensity. This was due to the typically high levels of anthocyanin pigments contained in both types of fruit [45]. The mean values of aroma intensity for all samples had a narrower range (3.46–4.77), with the control sample having a statistically significantly lower value and the samples of the coatings containing grape pomace having statistically significantly higher values. Grapes belong to the types of fruit with a typically more intense aroma. This intense aroma of grapes is generally one of the key factors for its higher sensory acceptability for consumers despite certain differences in the specific profile of volatile substances depending on the cultivar and the degree of maturity [46]. The mean values in the evaluation of the descriptor “surface roughness” ranged from 2.40 to 5.86, with several statistically significant differences. Samples GR_20, BL_20 and BL_5 showed statistically significantly higher roughness values. On the other hand, the samples with the two lowest concentrations of grape pomace (GR_5 and GR_10) had the lowest roughness. All analysed samples of edible coatings were characterised by a relatively low stickiness (2.11–3.14), with a statistically significantly lower stickiness documented only for sample GR_20 and with parsley pomace slightly increasing the level of stickiness (statistically significantly higher stickiness of samples PA_5 and PA_10 [3.14 and 3.05, respectively]). To be acceptable for consumer and commercial purposes, edible coatings should not be sticky; moreover, a smooth and glossy surface is desirable, especially for fruit coatings [8,47].

#### 3.1.2. Hedonic Analysis

Figure 2 and Table 4 show the results of the hedonic analysis of the edible coatings. Figure 2 shows a graph with the results of the main component analysis explaining the 98.62% variability by using two main components, one accounting for 80.06% and the other for 18.56% of the variability. The factor map of variables shows a close correlation between the overall evaluation and the descriptor of appearance. Table 4 shows the treated average values of descriptors obtained by the hedonic evaluation. The values highlighted in green represent a statistically significantly better rating (*p* < 0.05); the values highlighted in orange represent a statistically significantly worse rating. Both the location of each sample on the map of samples (Figure 2b) and Table 4 make it clear that the samples of edible coatings differed in many statistically significant (*p* < 0.05) aspects in the evaluation of pleasantness. The control sample, which did not contain any added plant pomace, achieved statistically significantly (*p* < 0.05) higher values in the descriptors pleasantness of appearance, pleasantness of aroma and overall evaluation. This may be due to the less intense colour and aroma of this sample, which, due to its neutral character, does not leave a disturbing impression, which is a key quality of an edible coating [48,49].

In the overall evaluation, another four samples containing parsley pomace in all concentrations and a sample containing 5% grape pomace achieved a statistically significantly (*p* < 0.05) better evaluation. Samples PA_5 and GR_5 also achieved a statistically significantly (*p* < 0.05) better evaluation for the descriptor pleasantness of appearance. The samples with the highest (20%) concentration of grape and blueberry pomace had the lowest overall evaluation. These two samples also had statistically significantly (*p* < 0.05) lower values of pleasantness of appearance and texture. Most likely, this was due to the high intensity of the colour, which prevented the achievement of a neutral character without any disturbing qualities.

The pleasantness of edible coating samples in individual descriptors was evaluated using a 9-point category scale: 1 = completely unpleasant; 5 = neutral; 9 = completely pleasant. Scores below 5 were considered as indicative of rejection of the sample due to unsatisfactory sensory quality [37,48]. From the point of view of the overall evaluation, only the samples with the highest (20%) concentrations of grape and blueberry pomace extract were rejected. However, in terms of individual descriptors, more samples were assessed as unsatisfactory (scoring less than 5).

Apart from the control sample, only two samples containing pomaces (PA_5, PA_10) had values higher than five for all hedonic descriptors at the same time, and were therefore not considered rejected in terms of sensory quality. However, the values obtained for all four hedonic descriptors ranged in narrow intervals, indicative of a relative similarity of the samples to each other in the absence of any extreme differences between the values. The overall evaluation values ranged in the interval of (4.49–6.32); this indicates that even the samples with the statistically significantly best evaluation were only slightly pleasant in the overall evaluation. However, some studies consider a value of 4 as the limit for product acceptability in terms of a sensory trait [38,50]. If this milder criterion is applied, all samples of edible coatings analysed in our study could be considered acceptable in terms of sensory quality. In a study performed by Gutiérrez and Álvarez [51], edible films based on native plantain flour (2%) with glycerol (1.5%), with added different concentrations of *Aloe vera* gel (0, 2, 4 and 6%) achieved lower values of overall acceptability (3.8–4.5 on a 0–10 scale). Karača et al. [52] developed alginate- and pectin-based edible films combining alginate and pectin with various proteins in immortelle (*Helichrysum italicum*) extract abundant in polyphenols. These films achieved a broader span of overall acceptability values (2–7.5 on a 1–9 scale) with the highest values for alginate- and pectin-based edible films with whey protein isolate and alginate-based film with hemp protein.

##### Probability of Purchasing Commodities in Edible Coating

The results of the analysis of the probability of purchasing different groups of food commodities packaged in the edible coatings are shown in Table 5. As most of the values were lower than 3, it is clear that the evaluators could not imagine the use of the analysed edible coatings for groups of commodities such as meat products, dairy products (cheese), baked products, or fruits and vegetables. Apart from the control sample, samples PA_5 and PA_10 had the highest values in terms of buying intention. The use of sample PA_5 for packaging vegetables and dairy products (cheese) and the use of sample PA_10 for packaging fruit and vegetables scored 3, indicating panellists’ indifference towards such products. Out of the samples containing plant extracts, the use of samples with 5% and 10% concentrations of parsley pomace was therefore the most conceivable for the panellists.

### 3.2. Results of Measuring Colour Parameters

**Figure 3:**
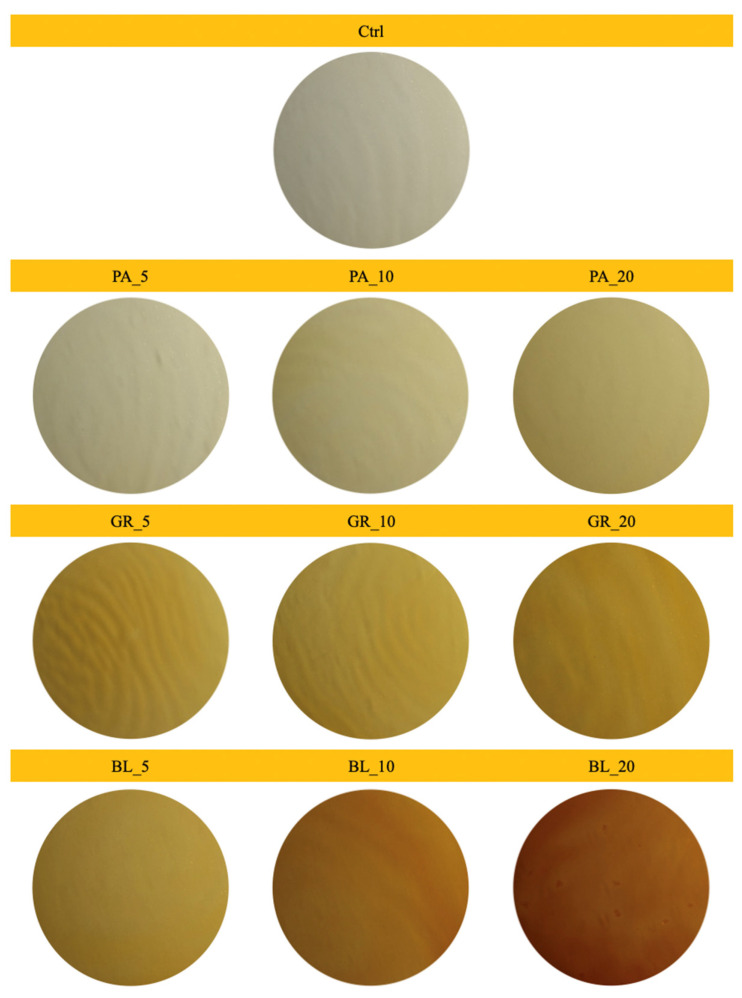
Digital images of edible coatings used for colour analysis (BL_5, edible coating with 5% of blueberry extract; BL_10, edible coating with 10% of blueberry extract; BL_20, edible coating with 20% of blueberry extract; GR_5, edible coating with 5% of red grape extract; GR_10, edible coating with 10% of red grape extract; GR_20, edible coating with 20% of red grape extract; PA_5, edible coating with 5% of parsley extract; PA_10, edible coating with 10% of parsley extract; PA_20, edible coating with 20% of parsley extract; Ctrl, control = edible coating without added extract).

Figure 3 contains images of edible coatings with added plant pomace. The results of the measurement of colour parameters (Table 6) demonstrate a statistically significant difference between almost all used additions and concentrations and the control sample without added plant pomace.

**Figure 4:**
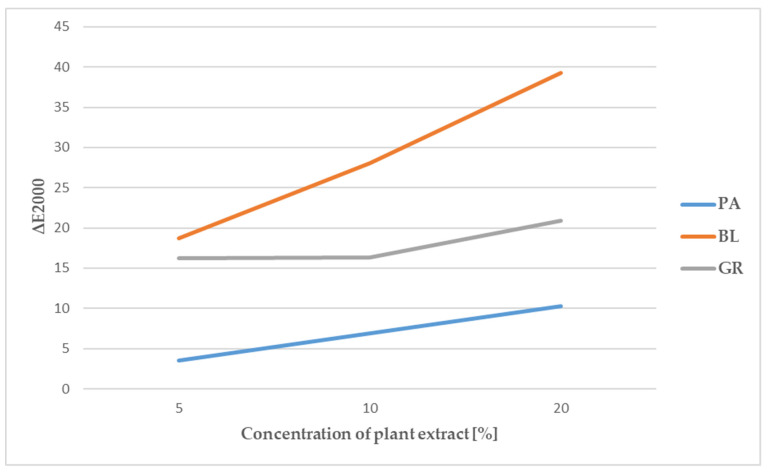
Colour difference between the control and the edible coating samples with added extracts (PA, parsley extract; BL, blueberry extract; GR, grape extract).

In the case of coatings that contained blueberry pomace, the value of a* increased with increasing concentration, whose positive values indicate an increasing proportion of the red component. Blueberries are rich in anthocyanins, coloured substances valued for their antioxidant capacity [52]. In an acidic environment, anthocyanins are red, but as the pH increases, their colour changes to blue [53]. The red colour of the coatings evaluated by us was therefore also partly due to the addition of lactic acid to the coatings. With the increasing concentration of the added blueberry pomace, the lightness value (L*) further decreased. A statistically significant difference (*p* < 0.01) between the samples with different concentrations of added blueberry pomace was demonstrated for the parameters L*, a* and b*, as was a statistically significant difference in the parameter ΔE (Figure 4), which describes the degree of difference between individual samples and the control without the addition of blueberry pomace. However, the lower stability of anthocyanins is a potential disadvantage of using blueberry pomace in edible coatings [54].

The evaluation of the colour parameters of the edible coatings with added parsley pomace demonstrated a statistically significant difference for most samples, both between individual samples and in comparison with the control. Only between the sample with the addition of 5% parsley pomace and the control sample was no statistically significant difference in the L* parameter demonstrated, as was between the sample with the addition of 20% of the same pomace and the control sample in the a* parameter. The positive values of the parameter b* correspond to the presence of the yellow colour [44]. In the case of samples of edible coatings containing parsley pomace, the parameters of their colour, in particular the parameter b*, were influenced by the presence of hydroxy derivatives of flavones and isoflavones, which belong to the yellow pigments. Apigenin, which is a natural bioactive component of parsley (*Petroselinum crispum* L.) with many therapeutic qualities, is also included in this group [55]. It is a flavonoid found in many species of fruit, vegetables, and herbs, but parsley is one of its most important sources [42].

In the case of edible coatings with added red grape pomace, no statistically significant difference in lightness (L*) was demonstrated between samples with the addition of 5 and 10% of the pomace (Table 6). As in the case of blueberries, the characteristic pigments contained in grape berries include anthocyanins, of which malvidin accounts for 68% [33,56,57]. As already mentioned, the colour of anthocyanins varies from red to blue depending on the pH [53]. The addition of grape pomace containing anthocyanins in combination with the used lactic acid led to an increase in the value of the parameter a*. In their study, Burin at el. [58] documented a robust positive correlation between the red colour and the total level of anthocyanins in grape juice.

In order to compare the control with samples with added pomace, the value of ΔE2000 was calculated. The results showed that the type and concentration of the pomace used had a statistically significant effect (*p* < 0.01) on the ΔE2000 values. In all cases, the value of ΔE2000 was greater than 1, which is the minimum value allowing the human eye to distinguish the difference [59]. The largest difference was obtained in samples with added blueberry and grape pomace. However, the edible coating samples containing grape pomace did not demonstrate a linear increase depending on the concentration of the added pomace. The lowest values of ΔE2000 were found in samples of edible coatings containing parsley pomace, with ΔE2000 ranging from 3.5 for the concentration of 5% to 10.3 for the concentration of 20% of the added pomace. 

However, the mere fact that the analysed samples of edible coatings with added plant pomace differed in colour from the control sample without added pomace does not automatically mean worse properties or lower usability of such coatings. Although the usual aim is to produce edible coatings that would least affect the sensory properties, such as the appearance and colour of the products, in certain specific cases, a darker colour may also be an advantage. Veiga-Santos et al. [60] believe that a darker colour can reduce the amount of light-induced oxidation of the product, and thus contribute to an improved protection of the food from oxidation, which could be particularly useful for food with a higher fat content. Another alternative solution may be the application of edible coatings with a more intense colour as secondary food coating [51]. Another possibility is the potential use of coatings with a more pronounced colour for enhancing the natural colour of similarly coloured food, thus assisting in their marketing apart from their primary protective purposes. Similarly, Benítez et al. [61] described a positive perception of edible coatings with aloe vera gel characterised by a greenish colour due to the contained chlorophyll when used to package kiwi slices.

### 3.3. Correlations between Sensory and Instrumental Colour Evaluations

Spearman’s correlation coefficient was used to express non-parametric correlations between sensory descriptors and colour parameters obtained by measuring instruments. According to Table 7, a number of statistically highly significant correlations (*p* < 0.01) were demonstrated between sensory descriptors related to the appearance and instrumentally assessed colour parameters of edible coatings with added plant pomace.

Hence, we may say that higher values of the L parameter suggest a higher sensory pleasantness of appearance, as does a lower degree of red/yellow colour of the edible coating. These instrumentally evaluated colour parameters accounted for a 21–46% variability of the sensory pleasantness of the appearance. The same trend was also followed by the correlation coefficients of the overall sensory pleasantness and the instrumentally evaluated colour parameters of the edible coatings; however, the values of the latter were lower and accounted for an 18–34% variability of the overall pleasantness in cases with a proven statistical significance. The values of instrumentally evaluated colour parameters of the edible coatings whose pleasantness was assessed by the sensory analysis as statistically significantly better are shown in Section 3.2.

### 3.4. Results of SEM Evaluation of the Surface of Edible Coatings

**Figure 5:**
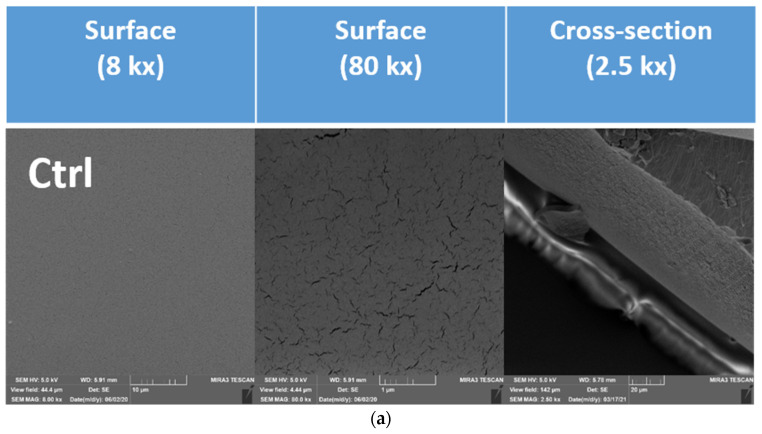

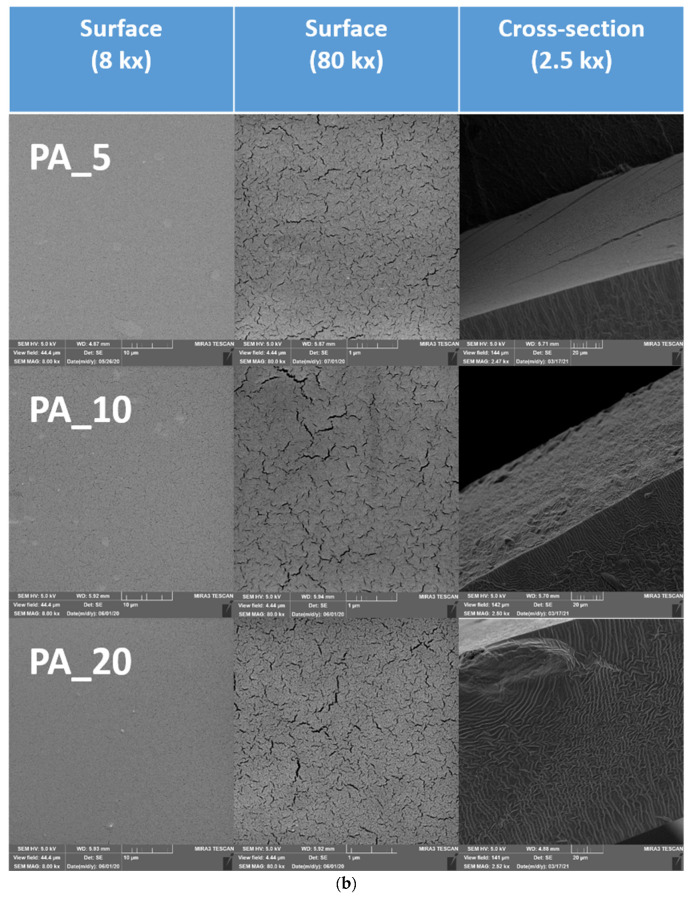

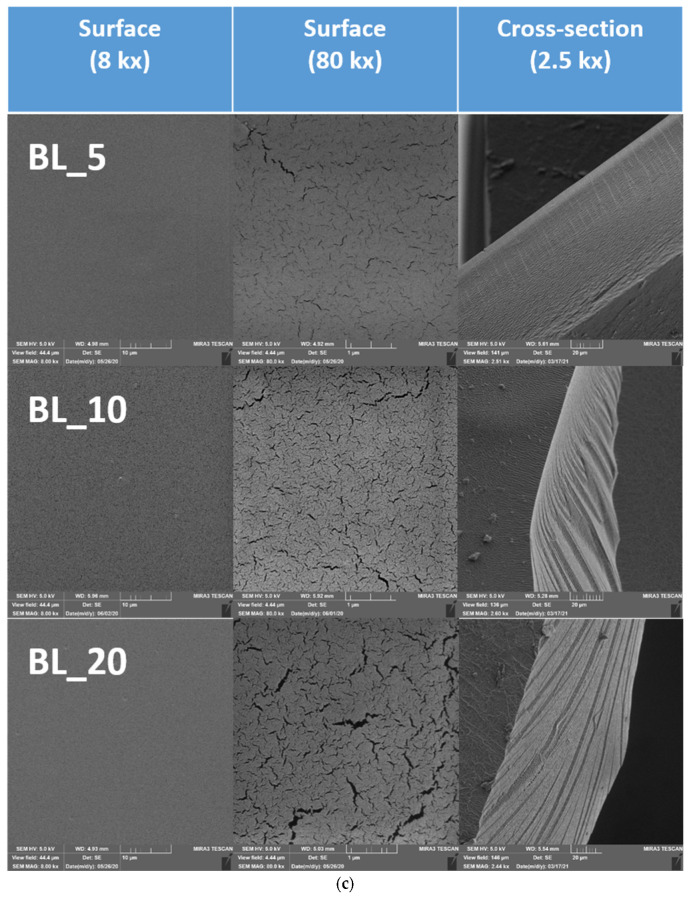

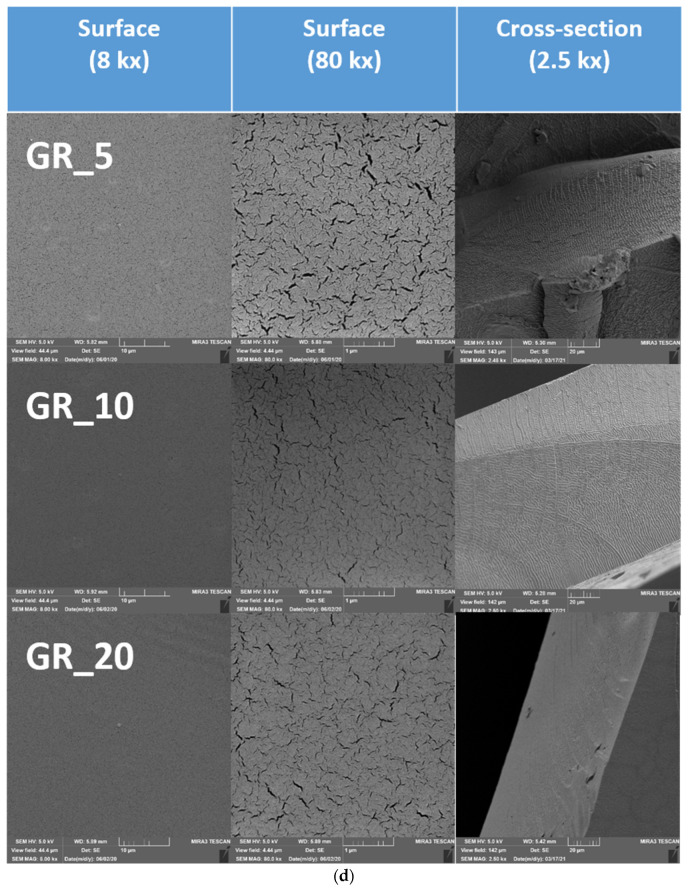
SEM images of edible coatings: surface images and cross-sections. (**a**) Ctrl, control = edible coating without added extract. (**b**) PA_5, edible coating with 5% of parsley extract; PA_10, edible coating with 10% of parsley extract; PA_20, edible coating with 20% of parsley extract. (**c**) BL_5, edible coating with 5% of blueberry extract; BL_10, edible coating with 10% of blueberry extract; BL_20, edible coating with 20% of blueberry extract. (**d**) GR_5, edible coating with 5% of red grape extract; GR_10, edible coating with 10% of red grape extract; GR_20, edible coating with 20% of red grape extract.

The scanning electron microscopy (SEM) technique was used to describe the microstructure, especially the occurrence of possible cracks, their area and the distance between these individual cracks. SEM images of the surfaces of the edible coatings as well as their cross-sections are represented in Figure 5a–d. 

**Figure 6:**
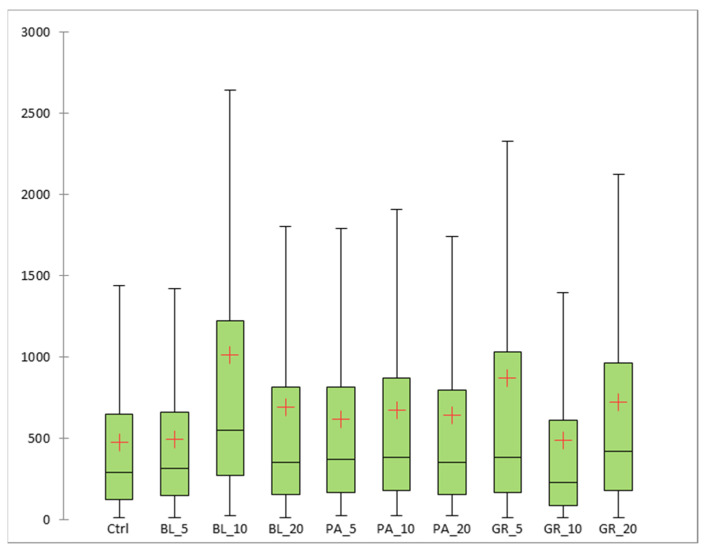
The results of SEM of edible coatings: the size of the cracks (fissure area) in the surface of the coatings [nm^2^] (BL_5, edible coating with 5% of blueberry extract; BL_10, edible coating with 10% of blueberry extract; BL_20, edible coating with 20% of blueberry extract; GR_5, edible coating with 5% of red grape extract; GR_10, edible coating with 10% of red grape extract; GR_20, edible coating with 20% of red grape extract; PA_5, edible coating with 5% of parsley extract; PA_10, edible coating with 10% of parsley extract; PA_20, edible coating with 20% of parsley extract; Ctrl, control = edible coating without added extract).

**Figure 7:**
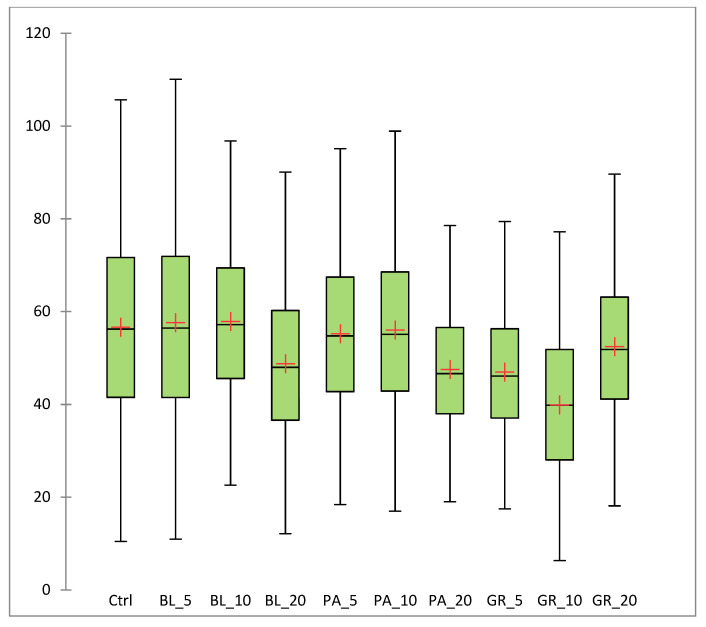
The results of SEM of edible coatings: the distance between cracks in the surface of the coatings (nm) (BL_5, edible coating with 5% of blueberry extract; BL_10, edible coating with 10% of blueberry extract; BL_20, edible coating with 20% of blueberry extract; GR_5, edible coating with 5% of red grape extract; GR_10, edible coating with 10% of red grape extract; GR_20, edible coating with 20% of red grape extract; PA_5, edible coating with 5% of parsley extract; PA_10, edible coating with 10% of parsley extract; PA_20, edible coating with 20% of parsley extract; Ctrl, control = edible coating without added extract).

Figure 6 documents the average size of the cracks in the surface of the formed gel. Figure 7 documents the distance between the cracks.

An analysis of the surface of the formed gel demonstrated the existence of differences between the coatings. In this study, the total area of the cracks and their mutual distance were used to measure the change of the surface. The cracks formed in the control sample had the smallest area (474.32 nm^2^ on average). The largest crack areas were confirmed in samples BL_10 (1014.51 nm^2^ on average) and GR_5 (868.22 nm^2^ on average). The smallest difference vs. the control was confirmed for samples BL_5 (494.33 nm^2^ on average), GR_10 (490.09 nm^2^ on average) and PA_5 (618.54 nm^2^ on average). A statistically significant difference (*p* < 0.05) in the area of cracks was documented between the control and all samples with added plant pomace. Our results did not confirm the effect of the addition of glycerol as a suitable plasticiser on the formation of the chitosan coating, as described by some authors [62,63]. This may be due to the smaller magnification (1–5 K) used in the mentioned studies. The formation of cracks was demonstrated in the samples analysed by us only at a magnification of 80 K; the changes were insignificant at smaller magnifications. Improvement of the plasticising properties of the coating could be achieved by the addition of another plasticiser, such as vegetable mucilage [64].

The distance between the cracks has a direct correlation to the total area R = 0.71 (*p* < 0.05). The larger the area, the smaller the distance between the cracks. A linear dependence of the increasing concentration on the distance between cracks was not confirmed. A distance between cracks (56.66 nm on average) was found in the control sample, without any significant difference as compared with samples BL_5 (57.61 nm on average), PA_5 (55.24 nm on average) and PA_10 (56.03 nm on average) (p˃0.05). In the other samples, a statistically significant difference (*p* < 0.05) vs. the control was observed in the distance between cracks.

Although the formation of cracks on the surface of the coating was proven at a magnification of 80 K, the cracks did not significantly interfere with the profile of the coating material. The transverse refractive indices did not document any cracks going through the whole layer (Figure 5). This means that the protective function of the edible coating was not disrupted by the added plant pomace. Khorram et al. [47] confirmed the presence of fractures and uneven surface at the addition of 3.5% Persian gum and 5% gelatine at as low a magnification as 500. It is therefore possible to assume that, unlike the addition of polymers, such as Persian gum and gelatine, the addition of pomace does not result in a change in the integrity of the full width of the formed coating but only on its surface.

**Figure 8:**
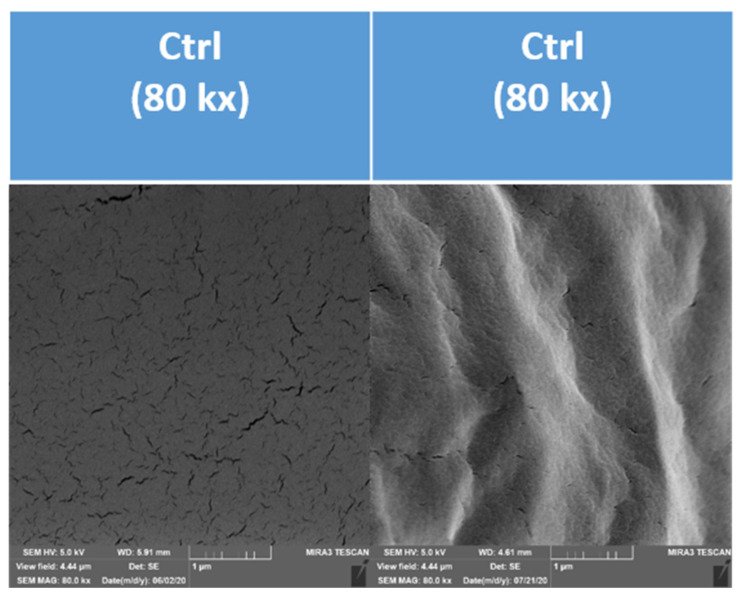
Effect of processing on the microscopic structure of the control sample of an edible coating. (**the left picture**) A coating formed on a strip. (**the right picture**) A coating formed outside a strip.

The obtained micrographs of the analysed coatings also showed the formation of an uneven surface during the handling of the samples (Figure 8). The information that the handling of the coating causes structural changes, manifested as the undulation of the surface, is an additional partial result of the study. A more suitable method for evaluation is the direct gel formation on the conductive target used in this study, which allows the assessment of the effect of the addition of pomace, rather than some accidental deformations caused during sample handling (transport, handling, refraction).

### 3.5. Biodegradability of Plastics by a Mixed Culture

**Figure 9:**
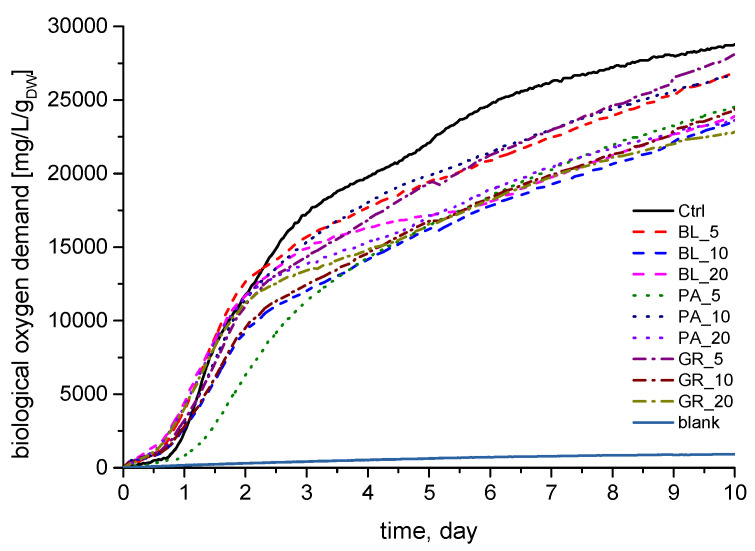
The course of respiratory activity of the tested biogas samples (the graph shows average values; *n* = 3).

Experiments with the biodegradability of bioplastics by a mixed culture have shown that all tested samples of bioplastics can serve as a substrate for a mixed culture of microorganisms. From the obtained values of biological oxygen demand (BOD) it is clear that the control sample, without the addition of pomace from plant materials, showed the highest BOD values (Figure 9, sample Ctrl). A lag phase, i.e., a period of 10 to 40 h necessary for adaptation of microorganisms after the addition of bioplastics, could be observed in all samples. The course of BOD was similar for all samples (except PA_5) for 60 h. Afterwards, a different biodegradability of the tested bioplastics began to show.

Compared to the control, all samples achieved lower final BOD values. The first group of samples achieved BOD values that were lower by 6.8% (BL_5), 7% (PA_10) and 3% (GR_5). The second group of samples achieved BOD values lower by 18% (BL_10), 17% (BL_20), 15% (PA_5), 18% (PA_20), 15% (GR_10) and 21% (GR_20) as compared to the control. Thanks to the good solubility of chitosan in water and the weak intermolecular bonds between chitosan and the plant pomace, relatively high values of biodegradation of the prepared bioplastics can be achieved. This has also been confirmed by other authors who have performed experiments with the biodegradation of chitosan-based bioplastics and plant pomaces [65].

## 4. Conclusions

The study indicated the influence of the examined plant extracts on coatings’ sensory properties, as well as on their barrier and biodegradability characteristics. The sensory quality of all edible coating samples analysed in our study was acceptable. In terms of overall pleasantness, the samples containing parsley pomace in all concentrations and a sample containing 5% grape pomace received the highest evaluations. As part of the evaluation of the probability of purchasing commodities in edible coating, the use of samples with 5% and 10% concentrations of parsley pomace was the most conceivable for the panellists. For all samples, the difference in colour from the control described by the ΔE2000 parameter was recognisable to the human eye. The lowest values were obtained for the sample containing 5% parsley pomace, the highest for the sample with 20% blueberry pomace. It was shown that the addition of plant pomace did not lead to a disruption of the protective function of the experimentally prepared edible coatings and that these coatings achieved relatively high values of biodegradation. The study gave clear directions for possible applications for coatings on various food commodities, including commercial-scale application. Further studies aimed at monitoring the interactions between coatings and packed food commodities and also coatings’ properties under different storage conditions and times need to be conducted. 

Our study suggests that coatings with incorporated grape, blueberry and in particular, parsley pomace extracts have the potential to be used on a commercial scale.

## Figures and Tables

**Table 2:** Composition of edible coatings.

Sample	Composition
Ctrl	1.5 g chitosan + 1% lactic acid + 0.75 mL glycerol
BL_5	1.5 g chitosan + 1% lactic acid + 5% blueberry extract + 0.75 mL glycerol
BL_10	1.5 g chitosan + 1% lactic acid + 10% blueberry extract + 0.75 mL glycerol
BL_20	1.5 g chitosan + 1% lactic acid + 20% blueberry extract + 0.75 mL glycerol
PA_5	1.5 g chitosan + 1% lactic acid + 5% parsley extract + 0.75 mL glycerol
PA_10	1.5 g chitosan + 1% lactic acid + 10% parsley extract + 0.75 mL glycerol
PA_20	1.5 g chitosan + 1% lactic acid + 20% parsley extract + 0.75 mL glycerol
GR_5	1.5 g chitosan + 1% lactic acid + 5% red grape extract + 0.75 mL glycerol
GR_10	1.5 g chitosan + 1% lactic acid + 10% red grape extract + 0.75 mL glycerol
GR_20	1.5 g chitosan + 1% lactic acid + 20% red grape extract + 0.75 mL glycerol

**Table 3:** Matrix with the *p*-values of the Hotelling’s T2 tests for each pair of edible film formulations (quantitative descriptive analysis).

	Bl_10	BL_20	BL_5	Ctrl	GR_10	GR_20	GR_5	PA_10	PA_20	PA_5
BL_10	1.00	0.06	*p* < 0.01	*p* < 0.01	*p* < 0.01	*p* < 0.01	*p* < 0.01	*p* < 0.05	*p* < 0.05	*p* < 0.01
BL_20	0.06	1.00	*p* < 0.05	*p* < 0.01	*p* < 0.01	0.08	*p* < 0.01	*p* < 0.01	*p* < 0.01	*p* < 0.01
BL_5	*p* < 0.01	*p* < 0.05	1.00	*p* < 0.01	*p* < 0.01	*p* < 0.05	*p* < 0.01	*p* < 0.01	*p* < 0.01	*p* < 0.01
Ctrl	*p* < 0.01	*p* < 0.01	*p* < 0.01	1.00	*p* < 0.01	*p* < 0.01	*p* < 0.01	0.14	*p* < 0.05	0.48
GR_10	*p* < 0.01	*p* < 0.01	*p* < 0.01	*p* < 0.01	1.00	*p* < 0.01	0.21	*p* < 0.01	*p* < 0.05	*p* < 0.01
GR_20	*p* < 0.01	0.08	*p* < 0.05	*p* < 0.01	*p* < 0.01	1.00	*p* < 0.01	*p* < 0.01	*p* < 0.01	*p* < 0.01
GR_5	*p* < 0.01	*p* < 0.01	*p* < 0.01	*p* < 0.01	0.21	*p* < 0.01	1.00	*p* < 0.01	*p* < 0.01	*p* < 0.01
PA_10	*p* < 0.05	*p* < 0.01	*p* < 0.01	0.14	*p* < 0.01	*p* < 0.01	*p* < 0.01	1.00	0.24	0.41
PA_20	*p* < 0.05	*p* < 0.01	*p* < 0.01	*p* < 0.05	*p* < 0.05	*p* < 0.01	*p* < 0.01	0.24	1.00	*p* < 0.05
PA_5	*p* < 0.01	*p* < 0.01	*p* < 0.01	0.48	*p* < 0.01	*p* < 0.01	*p* < 0.01	0.41	*p* < 0.05	1.00

* Statistically significant differences between the groups are emphasized with green colour. BL_5, edible film with 5% of blueberry extract; BL_10, edible film with 10% of blueberry extract; BL_20, edible film with 20% of blueberry extract; GR_5, edible film with 5% of red grape extract; GR_10, edible film with 10% of red grape extract; GR_20, edible film with 20% of red grape extract; PA_5, edible film with 5% of parsley extract; PA_10, edible film with 10% of parsley extract; PA_20, edible film with 20% of parsley extract; Ctrl, control = edible film without added extract.

**Table 4:** Adjusted mean of hedonic evaluation of edible coatings.

	Pleasantness of Texture	Pleasantness of Aroma	Pleasantness of Appearance	Overall Pleasantness
GR_20	5.031	4.456	4.048	4.488
BL_20	4.919	4.604	4.048	4.655
BL_5	5.012	4.771	4.770	5.062
BL_10	5.568	4.623	4.844	5.229
GR_10	6.568	4.642	4.918	5.414
PA_20	6.031	4.827	5.437	5.803
PA_10	5.808	5.067	5.474	5.766
PA_5	5.586	5.123	5.788	5.933
GR_5	6.827	4.827	5.529	5.859
Ctrl	6.049	5.549	6.511	6.322

BL_5, edible coating with 5% of blueberry extract; BL_10, edible coating with 10% of blueberry extract; BL_20, edible coating with 20% of blueberry extract; GR_5, edible coating with 5% of red grape extract; GR_10, edible coating with 10% of red grape extract; GR_20, edible coating with 20% of red grape extract; PA_5, edible coating with 5% of parsley extract; PA_10, edible coating with 10% of parsley extract; PA_20, edible coating with 20% of parsley extract; Ctrl, control = edible coating without added extract.

**Table 5:** Adjusted mean scores of the probability of purchasing a certain commodity in an edible coating.

	Meat Products	Vegetables	Fruit	Milk Products (Cheese)	Bakery Products
BL_10	1.655	2.121	2.117	1.600	1.467
BL_20	1.884	1.950	2.089	1.629	1.524
GR_20	2.055	1.836	1.974	2.000	1.524
GR_10	1.941	2.207	2.174	2.057	1.609
GR_5	2.084	2.264	2.231	1.943	1.724
BL_5	2.027	2.379	2.546	2.257	1.838
PA_20	2.141	2.721	2.517	2.315	1.867
PA_10	2.255	3.064	3.003	2.715	2.295
PA_5	2.255	3.121	2.946	3.143	2.381
Ctrl	2.284	3.35	3.403	3.229	2.495

BL_5, edible coating with 5% of blueberry extract; BL_10, edible coating with 10% of blueberry extract; BL_20, edible coating with 20% of blueberry extract; GR_5, edible coating with 5% of red grape extract; GR_10, edible coating with 10% of red grape extract; GR_20, edible coating with 20% of red grape extract; PA_5, edible coating with 5% of parsley extract; PA_10, edible coating with 10% of parsley extract; PA_20, edible coating with 20% of parsley extract; Ctrl, control = edible coating without added extract.

**Table 6:** Parameters of edible coating colour.

Concentration of Plant Extract				
**Blueberry Extract**	**5%**	**10%**	**20%**	**Control**
L*	53.443 ± 0.440	43.781 ± 1.132	33.124 ± 0.790	66.690 ± 0.441
a*	3.757 ± 0.087	14.324 ± 0.157	23.924 ± 0.221	−2.185 ± 0.066
b*	44.056 ± 0.177	46.980 ± 0.695	41.485 ± 0.589	12.773 ± 0.222
ΔE	18.672 ± 0.238	28.073 ± 0.756	39.297 ± 0.651	-
**Parsley Extract**	**5%**	**10%**	**20%**	**Control**
L*	66.998 ± 0.276 ^a^	65.683 ± 0.618	62.750 ± 0.286	66.690 ± 0.441 ^a^
a*	−2.863 ± 0.068	−3.281 ± 0.049	−2.243 ± 0.054 ^b^	−2.185 ± 0.066 ^b^
b*	18.627 ± 0.136	25.517 ± 0.174	32.124 ± 0.235	12.773 ± 0.222
ΔE	3.4858 ± 0.133	6.927 ± 0.200	10.253 ± 0.202	-
**Grape Extract**	**5%**	**10%**	**20%**	**Control**
L*	56.996 ± 0.616 ^c^	57.204 ± 0.721 ^c^	51.778 ± 0.788	66.690 ± 0.441
a*	1.541 ± 0.106	1.177 ± 0.133	6.178 ± 0.118	−2.185 ± 0.066
b*	43.223 ± 0.266	44.021 ± 0.319	48.256 ± 0.471	12.773 ± 0.222
ΔE	16.271 ± 0.229 ^d^	16.335 ± 0.244 ^d^	20.892 ± 0.350	-

Equal letters in the same row indicate no statistically significant differences (*p* < 0.01).

**Table 7:** Spearman’s correlation coefficients between sensory appearance descriptors and instrumental colour parameters of edible coating samples with added plant extracts.

	Colour Intensity	Pleasantness of Appearance	Overall Pleasantness
**Parsley extract**			
L*	−0.553 **	0.207 *	0.149
a*	−0.184 *	0.069	0.073
b*	0.674 **	−0.356 **	−0.153
**Grape extract**			
L*	−0.715 **	0.426 **	0.330 **
a*	0.638 **	−0.395 **	−0.281 **
b*	0.815 **	−0.428 **	−0.340 **
**Blueberry extract**			
L*	−0.857 **	0.461 **	0.316 **
a*	0.845 **	−0.459 **	−0.335 **
b*	0.381 **	−0.275 **	−0.178 *

* Asterisk next to the numerical value indicates statistically significant difference (*p* < 0.05); ** Two asterisks next to the numerical value indicate statistically very significant difference (*p* < 0.01).

## Data Availability

Not applicable.
